# Supplementary material for: Obstructive sleep apnea severity varies by season and environmental influences such as ambient temperature
Source: Commun Med (Lond). 2025 Jul 29;5:314. doi: 10.1038/s43856-025-01016-0 (PMC12307740; doi:10.1038/s43856-025-01016-0)
Supplement: Supplementary file 2 — Supplementary Material [file 43856_2025_1016_MOESM2_ESM.pdf]

Supplementary: Obstructive sleep apnea severity varies by season and environmental influences such as ambient temperature

Bastien Lechat[1#], Duc Phuc Nguyen[1], Kelly Sansom[1,2], Lucia Pinilla[1], Hannah Scott[1], Amy C. Reynolds[1], Andrew Vakulin[1], Jack Manners[1], Robert Adams[1], Jean-Louis Pepin[3], Pierre Escourrou[4], Peter Catcheside[1], Danny J. Eckert[1]

# Corresponding author

bastien.lechat@flinders.edu.au

Mark Oliphant Building, Level 2, Building A, 5 Laffer Drive, Bedford Park 5042

[1] Adelaide Institute for Sleep Health and FHMRI Sleep Health, College of Medicine and Public Health, Flinders University, Adelaide, Australia

[2] Centre for Healthy Ageing, Health Futures Institute, Murdoch University, Perth, Western Australia

[3] Univ. Grenoble Alpes, HP2 Laboratory, Inserm U-1300, CHU Grenoble Alpes, 38043 Grenoble, France.

[4] Centre Interdisciplinaire du Sommeil, Paris, France

Table S1: *Models to test for association between environmental factors and apnoea-hypopnoea-index.*

Figure*	Exposure	Model	Confounders	Covariates
4b	24h average temperature	Model 1	Day of year	Day of week
		Model 2	Model 1 + total cloud cover, relative humidity, air pollution, surface pressure	Day of week
4c	Air pollution	Model 1	Day of year	Day of week
		Model 2	Model 1 + total cloud cover, 24h average temperature, relative humidity, surface pressure	Day of week
4d	Relative humidity	Model 1	Day of year	Day of week
		Model 2	Model 1 + total cloud cover, 24h average temperature, air pollution, surface pressure	Day of week
4e	Surface Pressure	Model 1	Day of year	Day of week
		Model 2	Model 1 + total cloud cover + wind speed	Day of week
4f	Wind speed	Model 1	Day of year	Day of week
		Model 2	Model 1 + total cloud cover + surface pressure	Day of week
4g	Delta TST	Model 1	Day of year	Day of week
		Model 2	Model 1 + 24h average temperature, relative humidity, air pollution variation in TIB	Day of week
4h	Delta Sleep Timing	Model 1	Day of year	Day of week
		Model 2	Model 1 + 24h average temperature, relative humidity, air pollution + variation in TST	Day of week

*TST: total sleep time; TIB: time in bed; *results for a given variable are presented in Figure 4 of the main manuscript*

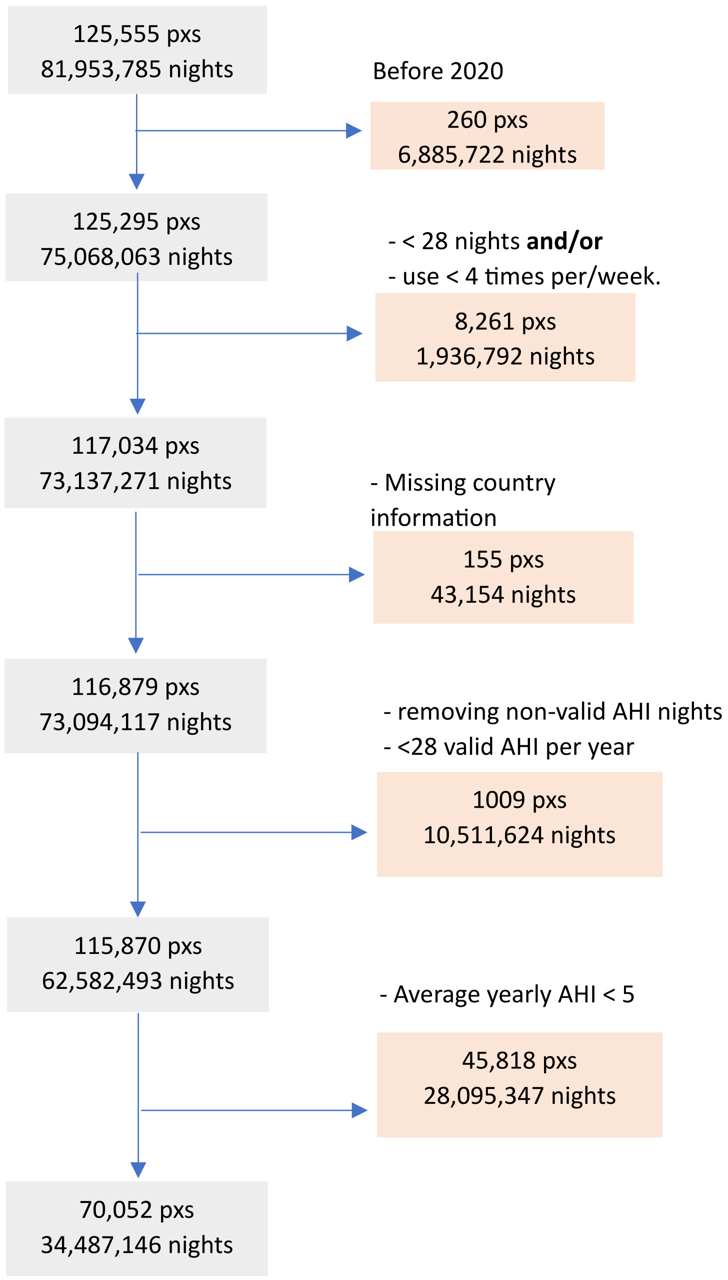

Figure S1: *Flowchart of user data. There were 125,555 users of the under-mattress sensors that used their devices between 2017 and September 2023. Data before January 2020 was removed given that the under-mattress device was validated in 2020[1]. Further inclusion criteria were at least 28 nights of data and an average use of the device of at least 4 times per week[2], 28 valid AHI per year, and an average yearly AHI of at least 5 events per hour. AHI: apnoea/hypopnoea index, Pxs: participants.*

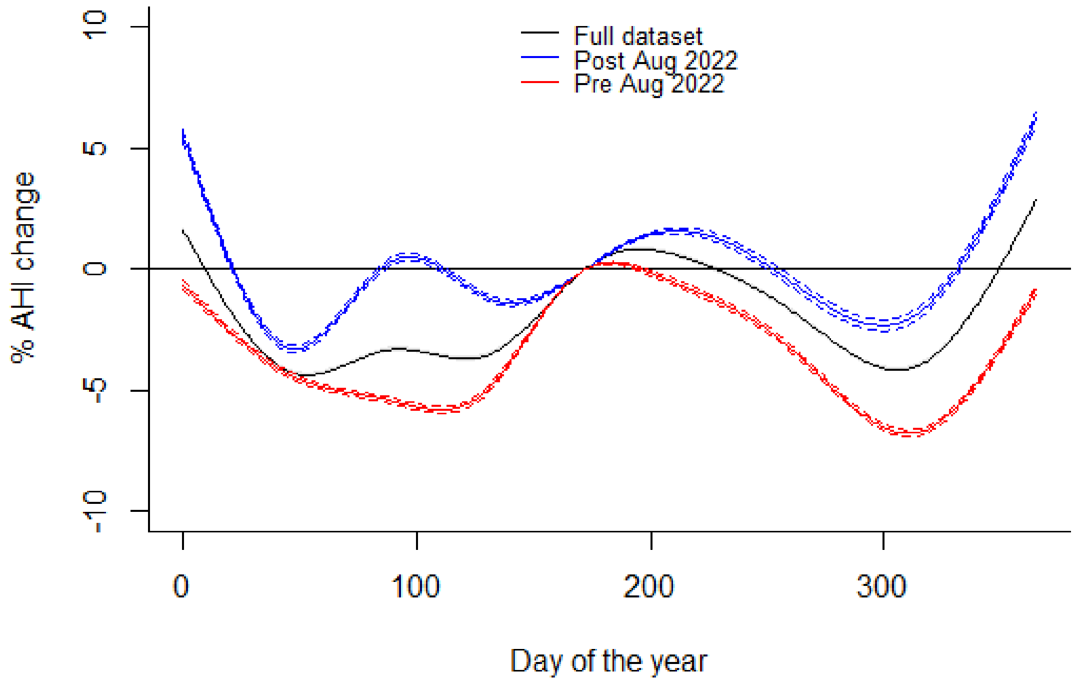

Figure S2: *Seasonal variation in the apnoea-hypopnoea index (AHI) pre-(red) and post (blue) COVID19 period. Black line represents is the exposure-response curve of the main manuscript. Analysis performed for the 30 to 90º latitude category only (representing 95.2% of the dataset). All lines represent estimated marginal means [and 95%CI] using the 21st of June as reference.*

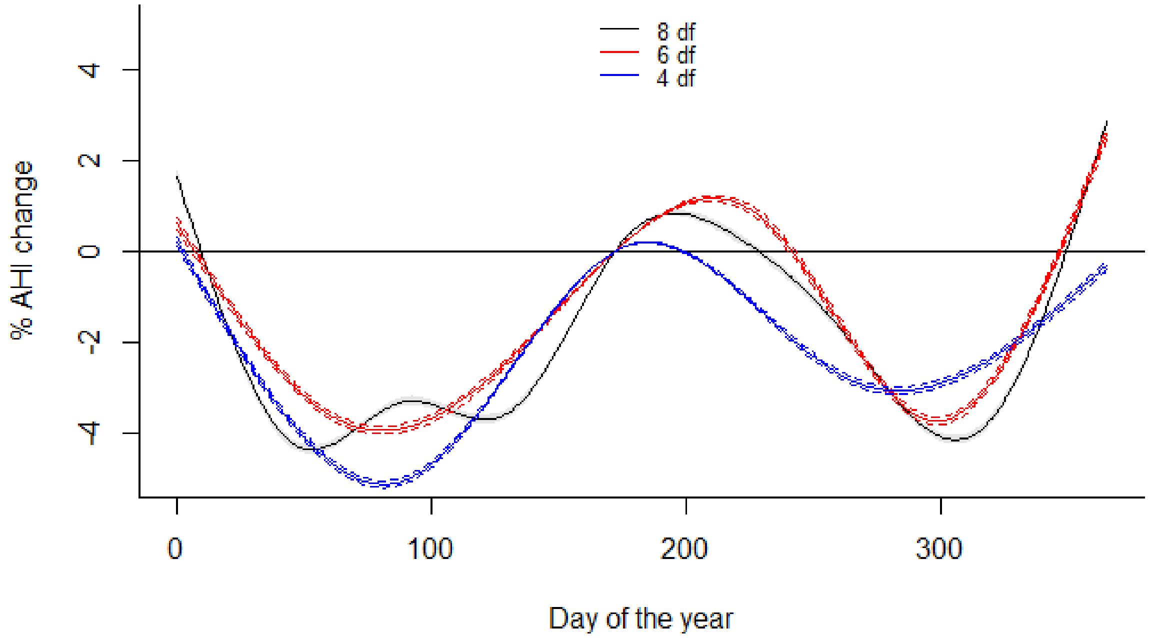

Figure S3: *Seasonal variation in the apnoea-hypopnoea index (AHI) using different degrees of freedom (df; 4 – blue, 6 – red, and 8 black – similar to the main analysis). The model with 8 degrees of freedom had the best model fit with the lowest Akaike information criterion. Analysis was performed for the 30 to 90º latitude category only (representing 95.2% of the dataset). All lines represent estimated marginal means [and 95%CI] using the 21st of June as reference.*

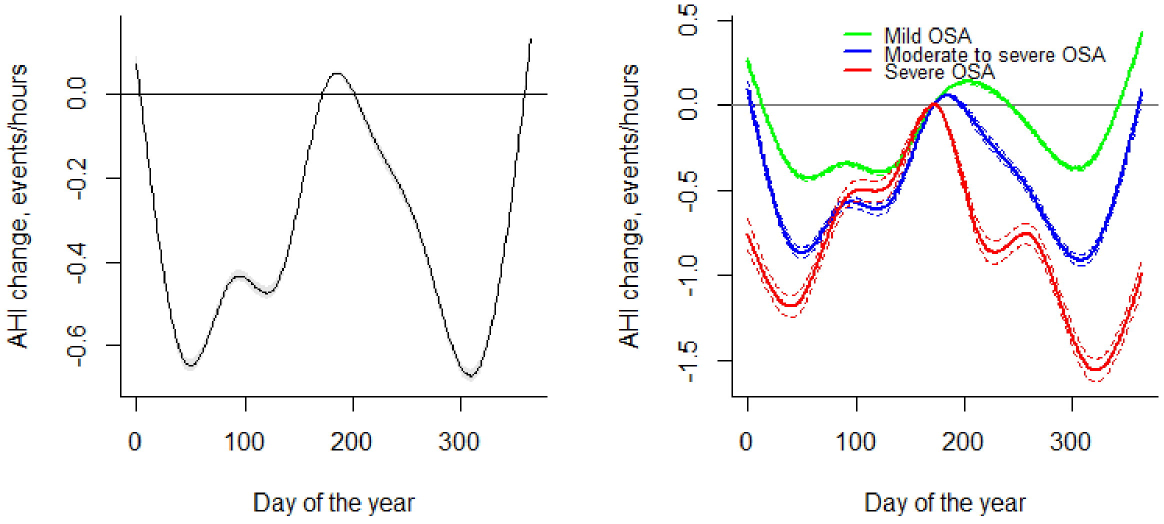

Figure S4: *Seasonal variation in the absolute apnoea-hypopnoea index (AHI) in the full dataset (left) and for different obstructive sleep apnoea (OSA) severity categories (right). Analysis performed for the 30 to 90º latitude category only (representing 95.2% of the dataset). All lines represent estimated marginal means [95%CI] using the 21st of June as reference. Absolute AHI change in events/hours was calculated as the difference in AHI between a given night and yearly average AHI for each participants and corresponds to* $AHI_{d,y,p} - \overline{AHI_{y,p}}$ *in Eq (1) of the manuscript. Exact peak-to-through coefficients (and 95%CI) and sample size for each group is available in Table S3 below.*

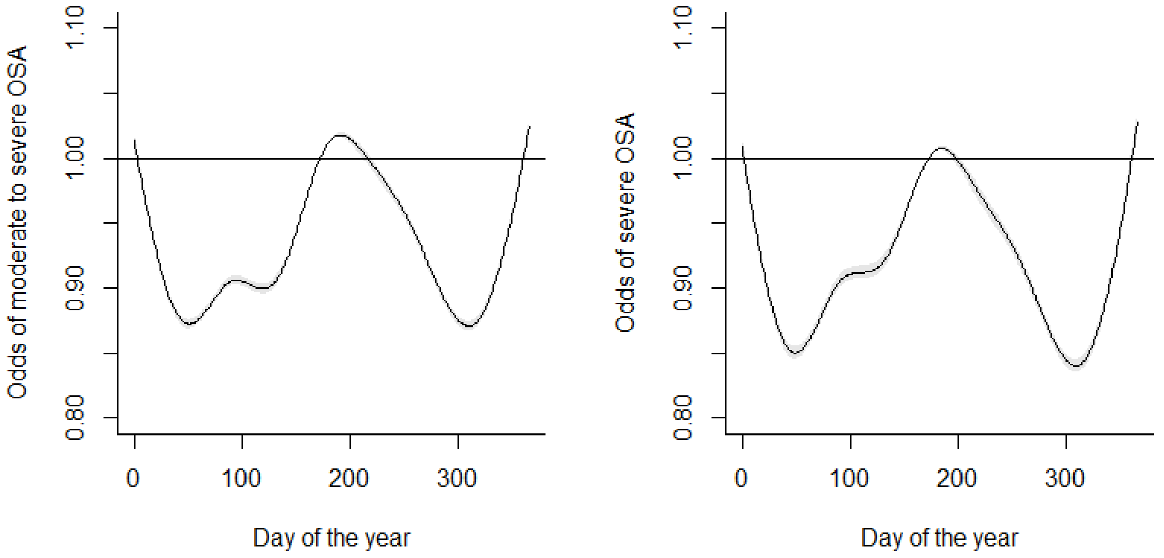

Figure S5: *Seasonal variation in the odds of moderate to severe obstructive sleep apnoea (OSA) (AHI≥ 15- left) and in the odds of severe OSA (AHI≥30 - right). Analysis performed for the 30 to 90º latitude category only (representing 95.2% of the dataset). Analysis performed for the 30 to 90º latitude category only (representing 95.2% of the dataset). All lines represent estimated marginal means [95%CI] using the 21st of June as reference.*

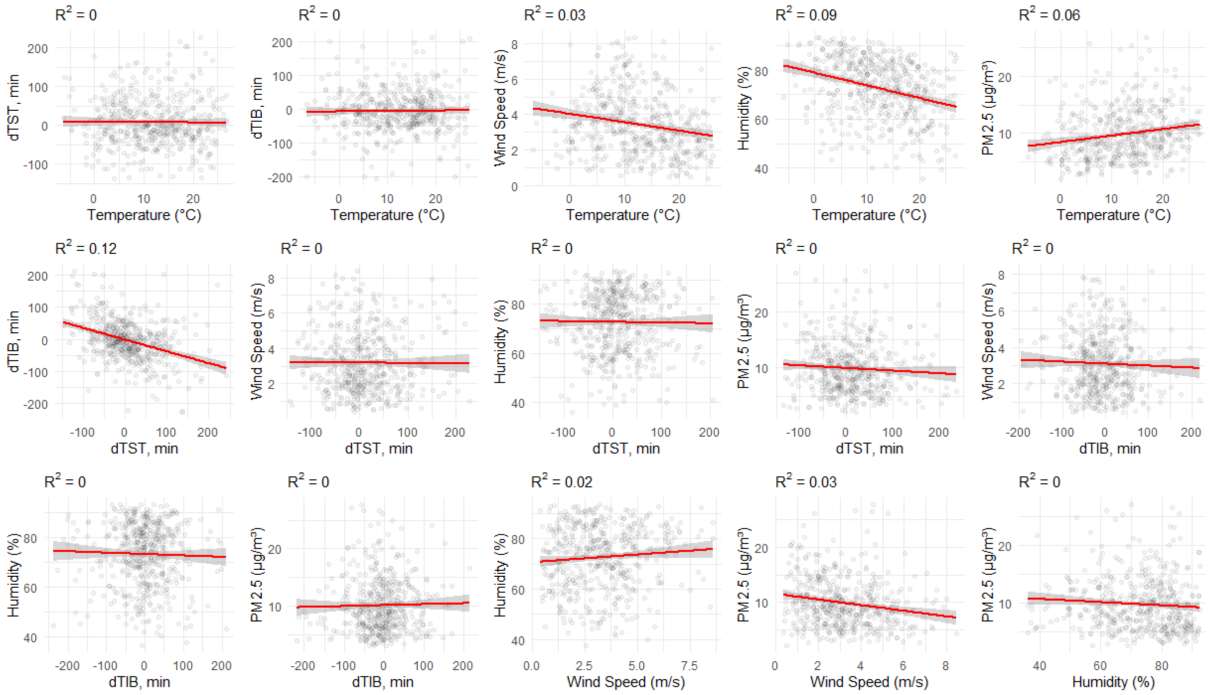

Figure S6: *Pairwise scatter plots and correlation coefficient (R^2) between environmental and sleep variables. Correlation coefficient and linear fit were determined on the full sample, but only N = 500 points were used for the scatter plots for clarity. dTST: Delta change in total sleep time; dTIB: Delta change in time in bed. All lines represent estimated marginal means and shaded area represents 95%CI.*

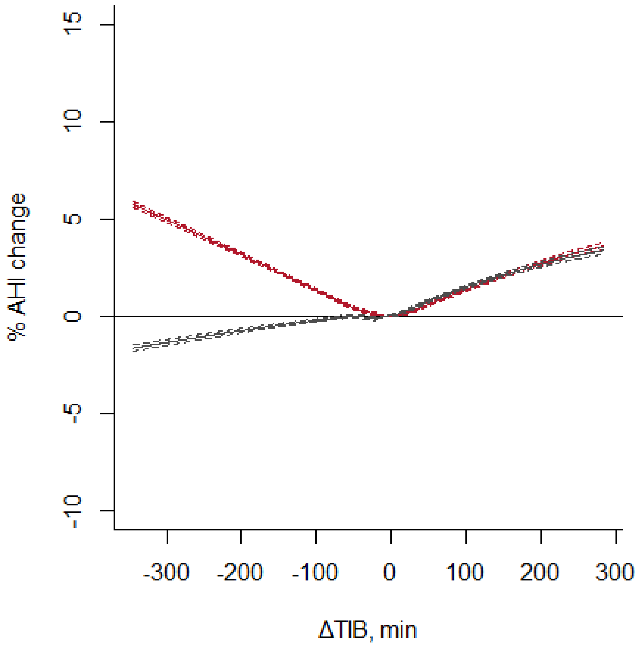

Figure S7: *Association between apnoea-hypopnoea-index (AHI) and variation in sleep timing defined as the difference between a given nighttime in bed (TIB) with the yearly TIB average in minutes. Red model is an adjusted model without adjustment for variation in total sleep time and black is the fully adjusted model with adjustment for variation in total sleep time. Note that once adjusting for variation in total sleep time, the association between less than normal time-in-bed with AHI change disappear.*

Table S2: *Association between a decrease in Δtotal sleep time (ΔTST; 5th vs. 50th) and apnoea/hypopnea index, expressed as a % of yearly mean. Δ total sleep time was defined as the variation in sleep duration from the yearly mean.*

Country	ΔTST (5th vs. 50th)
**Netherlands**	-0.7 (-1.1, -0.2)
**Denmark**	0.3 (-0.5, 1.0)
**Ireland**	2.2 (1.1, 3.4)
**United Kingdom**	0.8 (0.5, 1.0)
**Hungary**	-0.4 (-1.5, 0.7)
**Romania**	0.1 (-1.4, 1.5)
**Japan**	6.6 (6.1, 7.1)
**Norway**	-2.3 (-3.1, -1.4)
**Germany**	0.8 (0.7, 1.0)
**Italy**	2.5 (1.9, 3.0)
**Portugal**	-2.4 (-3.4, -1.4)
**Australia**	-0.5 (-1.1, 0.0)
**Belgium**	-1.0 (-1.5, -0.4)
**Poland**	-1.3 (-2.2, -0.3)
**Sweden**	-1.4 (-2.0, -0.8)
**Czech Republic**	-2.5 (-3.7, -1.2)
**Switzerland**	-0.7 (-1.0, -0.3)
**Spain**	1.1 (0.5, 1.8)
**France**	0.3 (0.1, 0.5)
**Finland**	-2.9 (-3.5, -2.3)
**Austria**	2.9 (2.3, 3.5)
**United States**	2.6 (2.5, 2.8)
**Canada**	1.3 (0.9, 1.8)

Table S3: *Sample size, coefficient and 95% CI of the seasonal variation in the absolute apnoea-hypopnoea index (AHI) in the full dataset and for different OSA severity categories from Figure S6. Analysis performed for the 30 to 90⁰ latitude category only (representing 95.2% of the dataset).*

	N[#]	*Mean (SD) AHI*	*Seasonal AHI change**
All	*68290*	*18.0 (14.0)*	*0.80 [0.78, 0.82]*
Mild OSA	*49057*	*10.4 (4.2)*	*0.85 [0.83, 0.87]*
Moderate to severe OSA	*25149*	*23.1 (6.0)*	*1.00 [0.96, 1.05]*
Severe OSA	*11775*	*46.3 (13.7)*	*1.56 [1.49, 1.62]*

*[#]Average AHI severity is calculated per year for each participants, hence the sum of the different OSA severity categories may be higher than the total amount of participants (some participant have mild OSA one year, moderate to severe OSA the next) *Seasonal effect is summarized using the mean (95%CI) difference between the peak and the trough of the seasonal variation in OSA severity.*

Top 12 countries:

Each of the Figures below presents seasonal variation in apnoea-hypopnoea index (AHI) for participants in the top 12 countries with the most users. The % increase in AHI was calculated as the difference between the AHI on a given night versus the yearly average, expressed as % of yearly average (see more details in the results section). For each country, we provide a description of the data sample with mean age, number of participants, and other relevant covariates.

Figure SC1: United States........ 11
Figure SC2: Germany. 12
Figure SC3: France. 13
Figure SC4: United Kingdom. 14
Figure SC5: Switzerland. 15
Figure SC6: Japan. 16
Figure SC7: Netherlands. 17
Figure SC8: Canada. 18
Figure SC9: Italy. 19
Figure SC10: Finland. 20
Figure SC11: Belgium. 21
Figure SC12: Sweden. 22

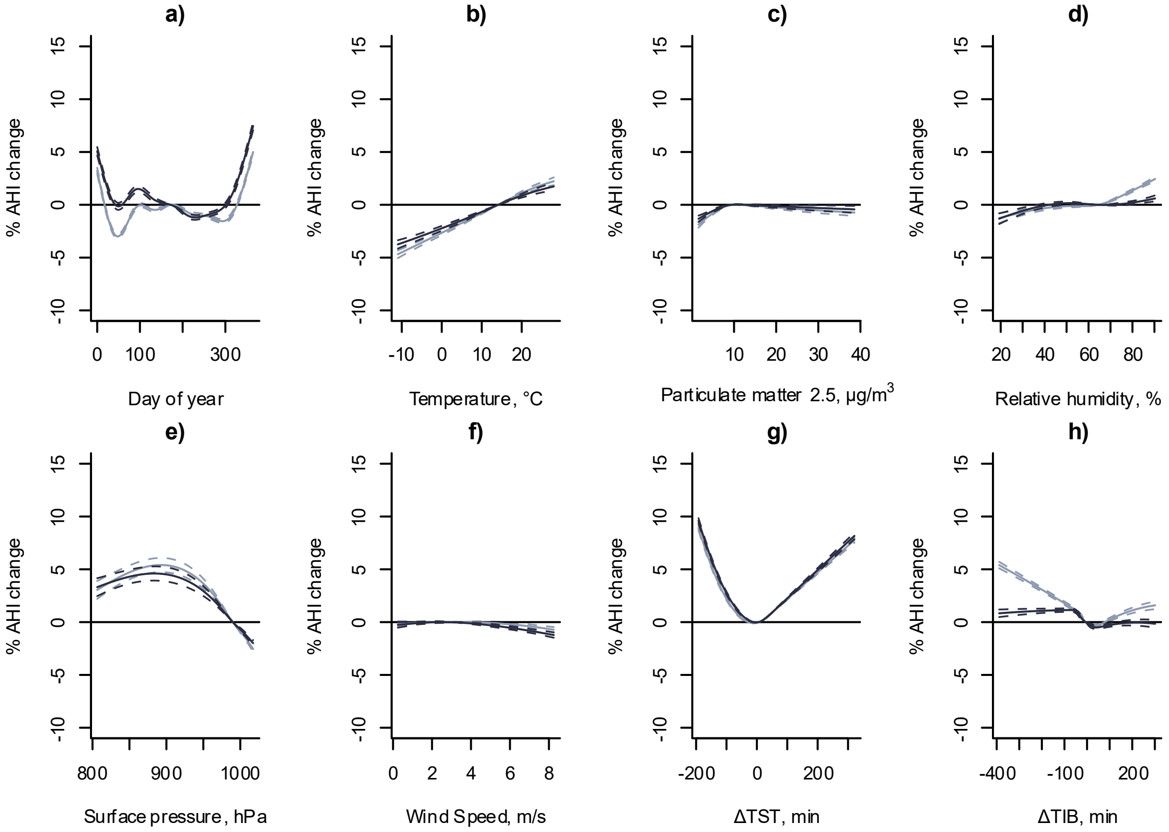

Figure SC1: United States.

Description: *Associations of different environmental and sleep-specific factors with seasonal variation in the apnoea-hypopnoea-index (AHI) for unadjusted (blue) and fully adjusted models (black). a) Day of the year (21st of June as reference), b) 24h average temperature, c) density of particulate matter with diameter of less than 2.5um d) relative humidity e) surface pressure, f) wind speed g) difference between a given night total sleep time (TST) with the yearly TST average (ΔTST) in minutes and h) similar to g) but for time in bed (ΔTIB). All graphs represent estimated marginal means using the 50th percentiles as the reference value (except for a)), and the x-axis limits were set as the 1st percentile and the 99th percentiles.*

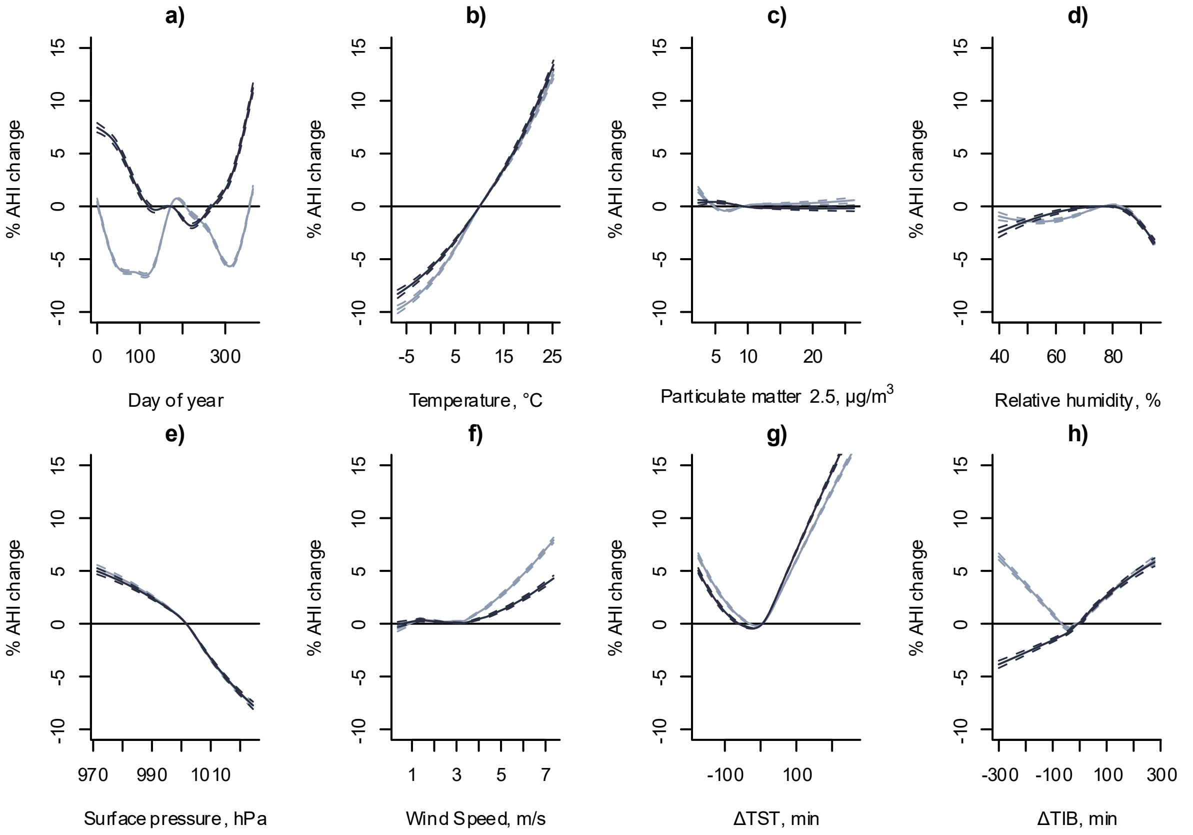

Figure SC2: Germany.

Description: *Associations of different environmental and sleep-specific factors with seasonal variation in the apnoea-hypopnoea-index (AHI) for unadjusted (blue) and fully adjusted models (black). a) Day of the year (21st of June as reference), b) 24h average temperature, c) density of particulate matter with diameter of less than 2.5um d) relative humidity e) surface pressure, f) wind speed g) difference between a given night total sleep time (TST) with the yearly TST average (ΔTST) in minutes and h) similar to g) but for time in bed (ΔTIB). All graphs represent estimated marginal means using the 50th percentiles as the reference value (except for a)), and the x-axis limits were set as the 1st percentile and the 99th percentiles.*

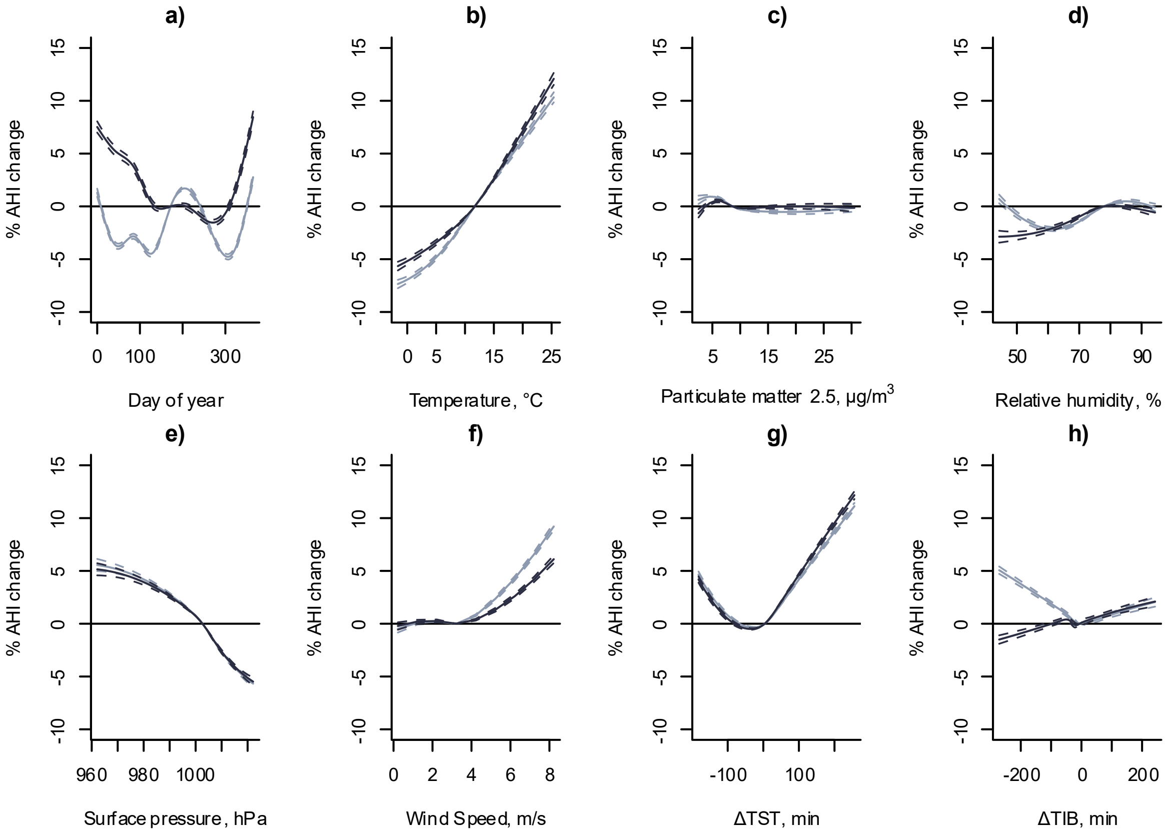

Figure SC3: France.

Model description: *Associations of different environmental and sleep-specific factors with seasonal variation in the apnoea-hypopnoea-index (AHI) for unadjusted (blue) and fully adjusted models (black). a) Day of the year (21st of June as reference), b) 24h average temperature, c) density of particulate matter with diameter of less than 2.5um d) relative humidity e) surface pressure, f) wind speed g) difference between a given night total sleep time (TST) with the yearly TST average (ΔTST) in minutes and h) similar to g) but for time in bed (ΔTIB). All graphs represent estimated marginal means using the 50th percentiles as the reference value (except for a)), and the x-axis limits were set as the 1st percentile and the 99th percentiles.*

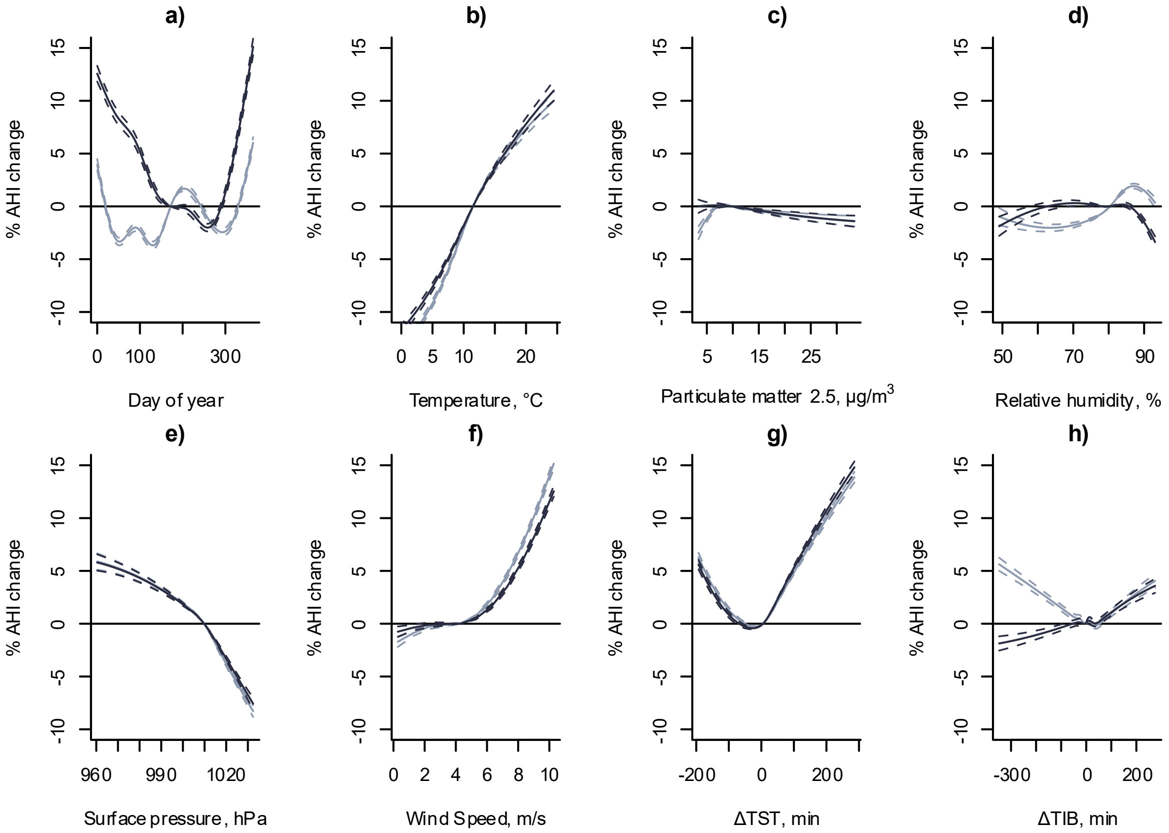

Figure SC4: United Kingdom.

Model description: *Associations of different environmental and sleep-specific factors with seasonal variation in the apnoea-hypopnoea-index (AHI) for unadjusted (blue) and fully adjusted models (black). a) Day of the year (21st of June as reference), b) 24h average temperature, c) density of particulate matter with diameter of less than 2.5um d) relative humidity e) surface pressure, f) wind speed g) difference between a given night total sleep time (TST) with the yearly TST average (ΔTST) in minutes and h) similar to g) but for time in bed (ΔTIB). All graphs represent estimated marginal means using the 50th percentiles as the reference value (except for a)), and the x-axis limits were set as the 1st percentile and the 99th percentiles.*

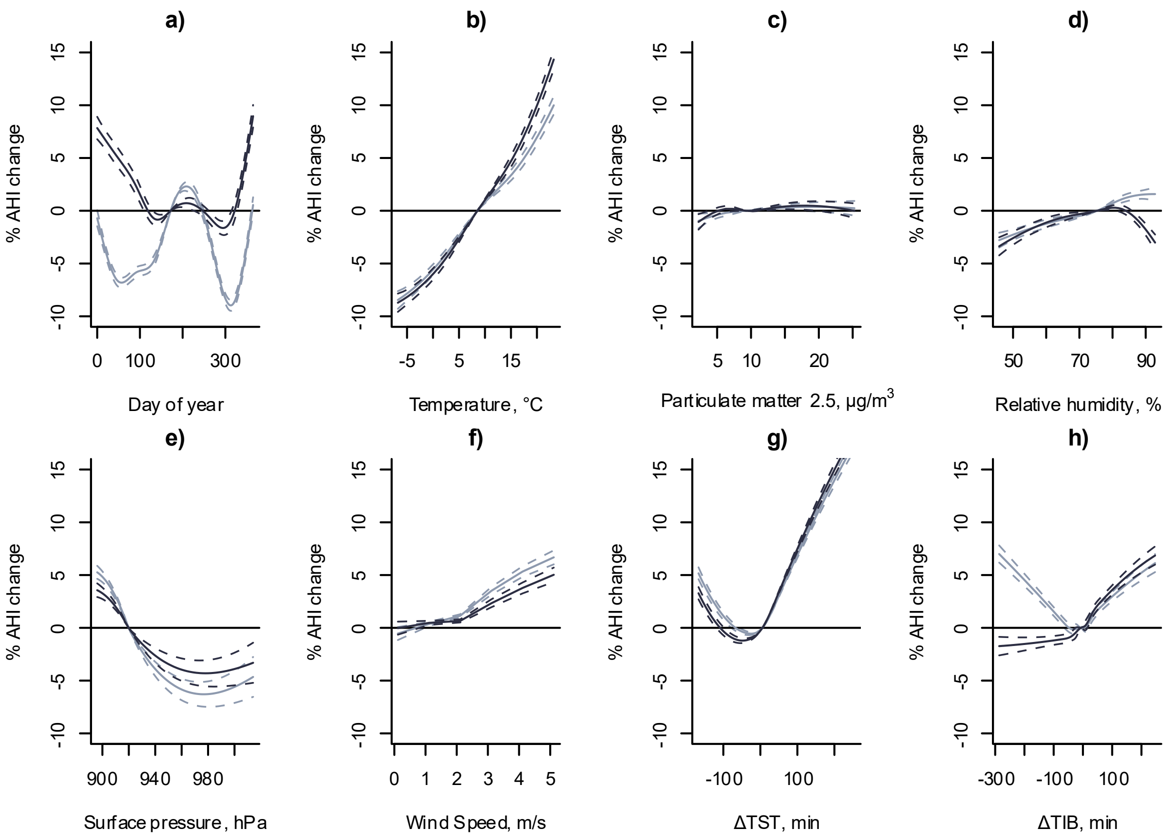

Figure SC5: Switzerland.

Model description: *Associations of different environmental and sleep-specific factors with seasonal variation in the apnoea-hypopnoea-index (AHI) for unadjusted (blue) and fully adjusted models (black). a) Day of the year (21st of June as reference), b) 24h average temperature, c) density of particulate matter with diameter of less than 2.5um d) relative humidity e) surface pressure, f) wind speed g) difference between a given night total sleep time (TST) with the yearly TST average (ΔTST) in minutes and h) similar to g) but for time in bed (ΔTIB). All graphs represent estimated marginal means using the 50th percentiles as the reference value (except for a)), and the x-axis limits were set as the 1st percentile and the 99th percentiles.*

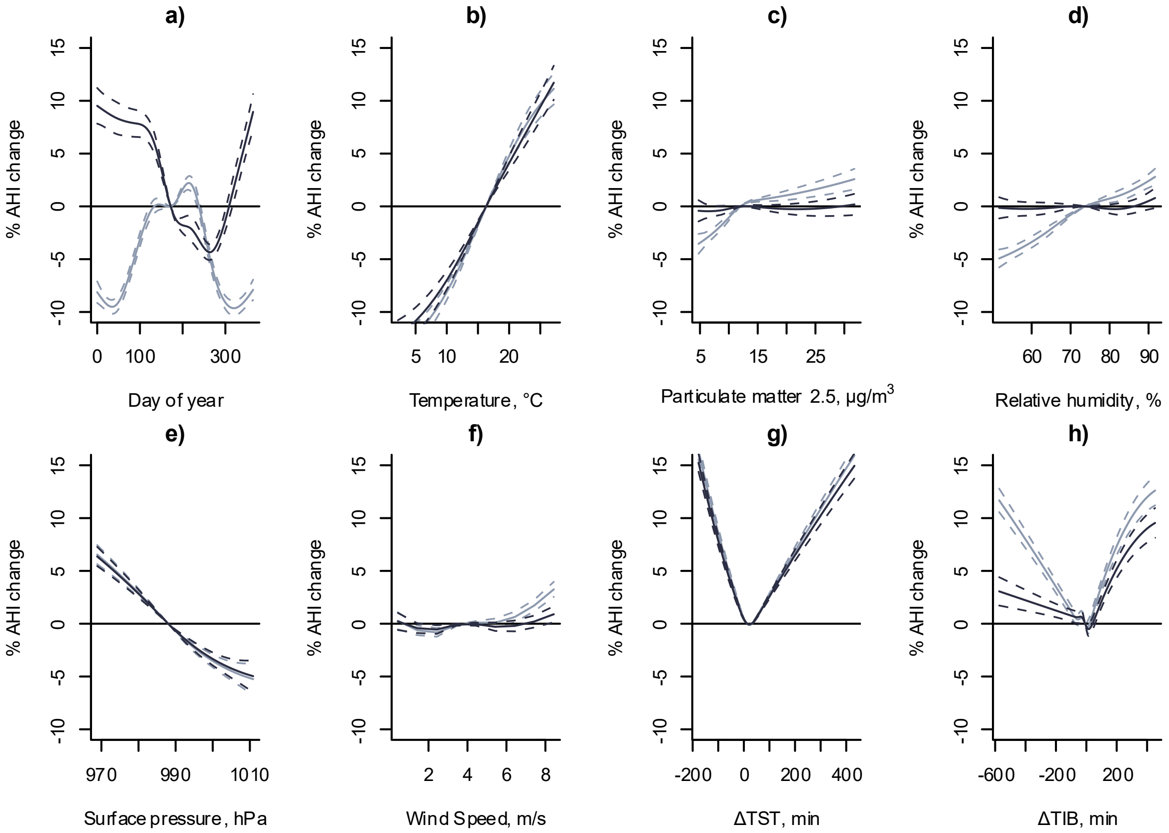

Figure SC6: Japan.

Model description: *Associations of different environmental and sleep-specific factors with seasonal variation in the apnoea-hypopnoea-index (AHI) for unadjusted (blue) and fully adjusted models (black). a) Day of the year (21st of June as reference), b) 24h average temperature, c) density of particulate matter with diameter of less than 2.5um d) relative humidity e) surface pressure, f) wind speed g) difference between a given night total sleep time (TST) with the yearly TST average (ΔTST) in minutes and h) similar to g) but for time in bed (ΔTIB). All graphs represent estimated marginal means using the 50th percentiles as the reference value (except for a)), and the x-axis limits were set as the 1st percentile and the 99th percentiles.*

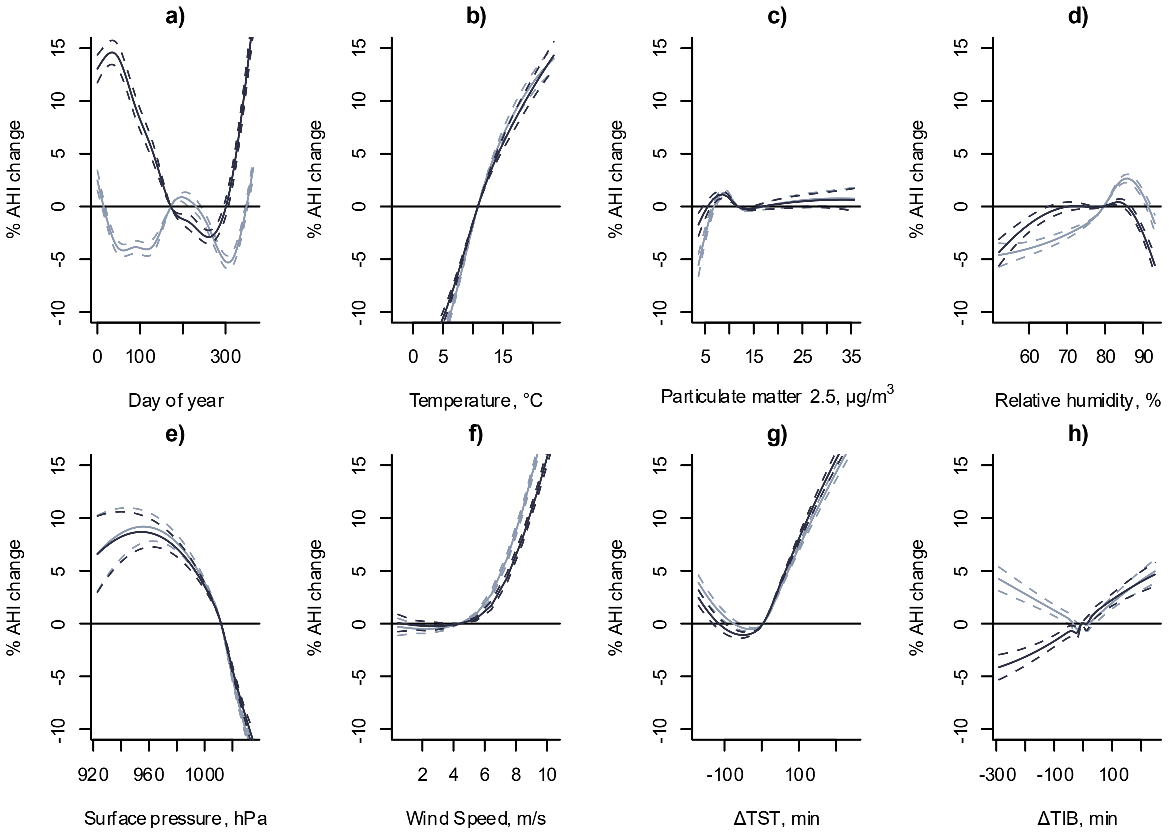

Figure SC7: Netherlands.

Model description: *Associations of different environmental and sleep-specific factors with seasonal variation in the apnoea-hypopnoea-index (AHI) for unadjusted (blue) and fully adjusted models (black). a) Day of the year (21st of June as reference), b) 24h average temperature, c) density of particulate matter with diameter of less than 2.5um d) relative humidity e) surface pressure, f) wind speed g) difference between a given night total sleep time (TST) with the yearly TST average (ΔTST) in minutes and h) similar to g) but for time in bed (ΔTIB). All graphs represent estimated marginal means using the 50th percentiles as the reference value (except for a)), and the x-axis limits were set as the 1st percentile and the 99th percentiles.*

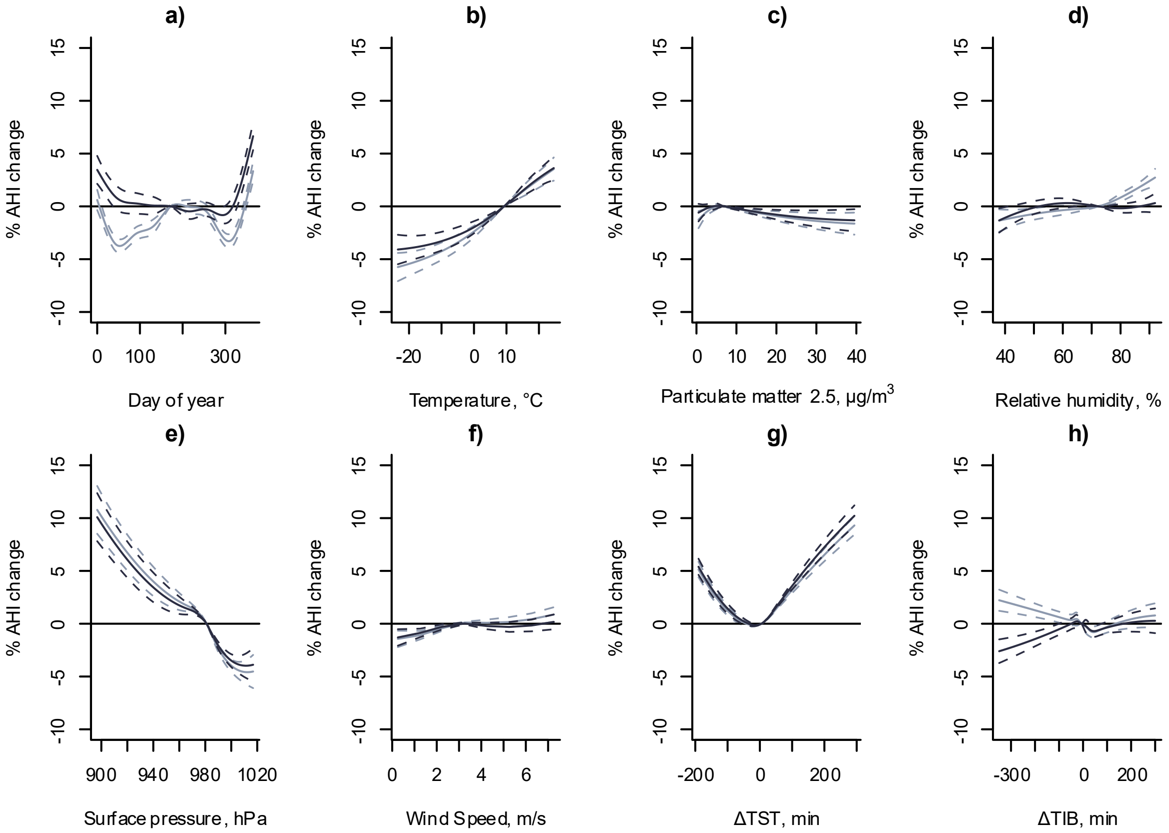

Figure SC8: Canada.

Model description: *Associations of different environmental and sleep-specific factors with seasonal variation in the apnoea-hypopnoea-index (AHI) for unadjusted (blue) and fully adjusted models (black). a) Day of the year (21st of June as reference), b) 24h average temperature, c) density of particulate matter with diameter of less than 2.5um d) relative humidity e) surface pressure, f) wind speed g) difference between a given night total sleep time (TST) with the yearly TST average (ΔTST) in minutes and h) similar to g) but for time in bed (ΔTIB). All graphs represent estimated marginal means using the 50th percentiles as the reference value (except for a)), and the x-axis limits were set as the 1st percentile and the 99th percentiles.*

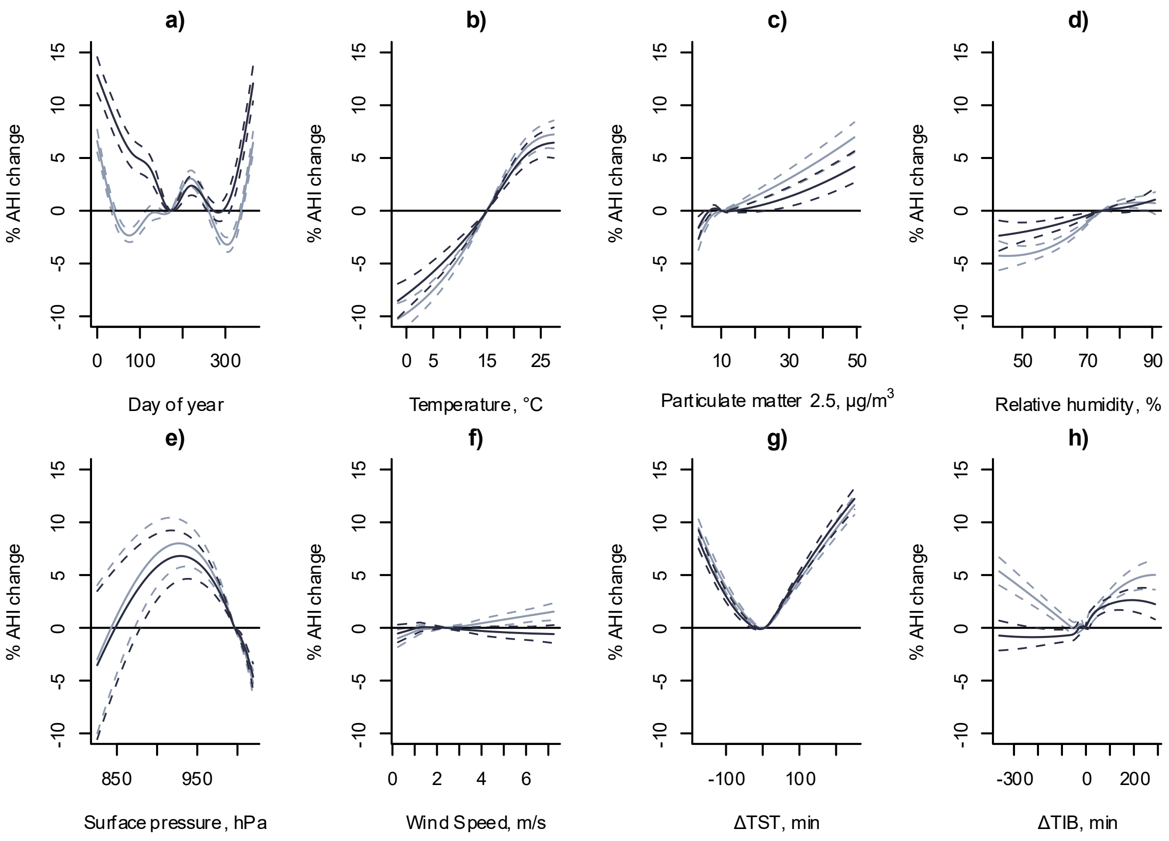

Figure SC9: Italy.

Model description: *Associations of different environmental and sleep-specific factors with seasonal variation in the apnoea-hypopnoea-index (AHI) for unadjusted (blue) and fully adjusted models (black). a) Day of the year (21st of June as reference), b) 24h average temperature, c) density of particulate matter with diameter of less than 2.5um d) relative humidity e) surface pressure, f) wind speed g) difference between a given night total sleep time (TST) with the yearly TST average (ΔTST) in minutes and h) similar to g) but for time in bed (ΔTIB). All graphs represent estimated marginal means using the 50th percentiles as the reference value (except for a)), and the x-axis limits were set as the 1st percentile and the 99th percentiles.*

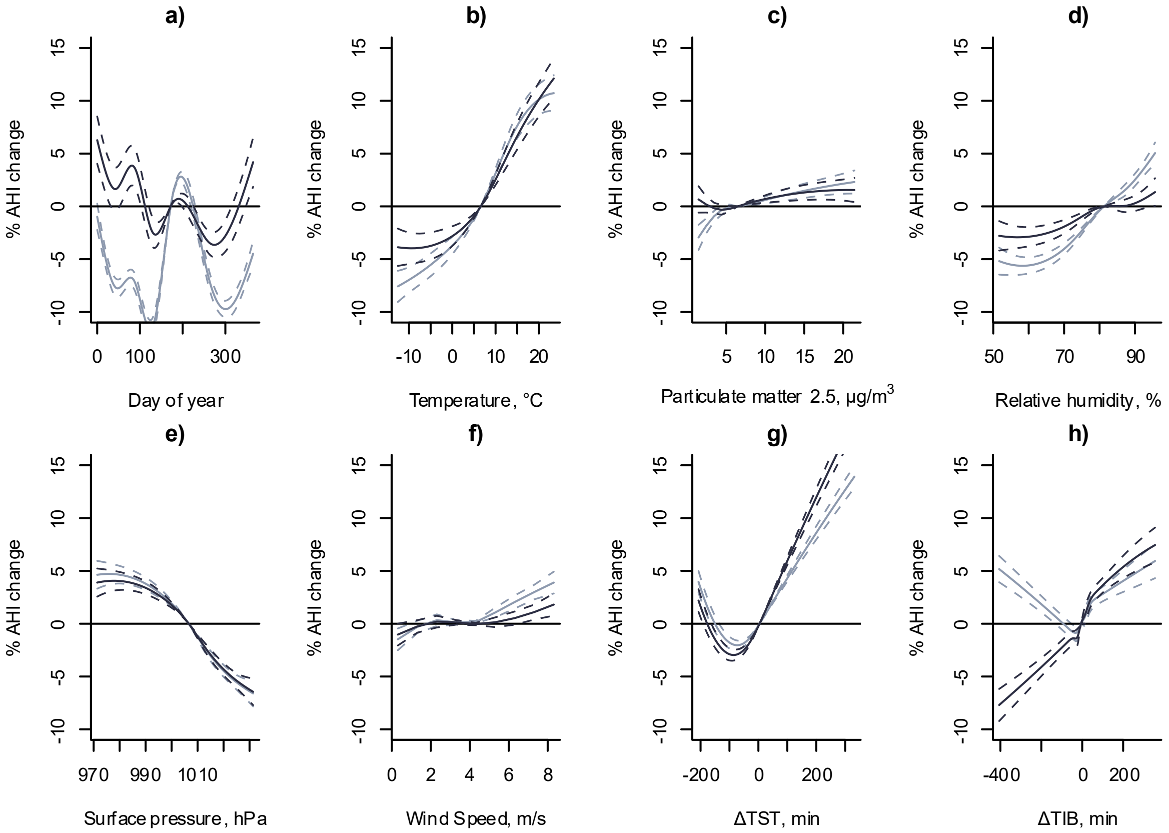

Figure SC10: Finland.

Model description: *Associations of different environmental and sleep-specific factors with seasonal variation in the apnoea-hypopnoea-index (AHI) for unadjusted (blue) and fully adjusted models (black). a) Day of the year (21st of June as reference), b) 24h average temperature, c) density of particulate matter with diameter of less than 2.5um d) relative humidity e) surface pressure, f) wind speed g) difference between a given night total sleep time (TST) with the yearly TST average (ΔTST) in minutes and h) similar to g) but for time in bed (ΔTIB). All graphs represent estimated marginal means using the 50th percentiles as the reference value (except for a)), and the x-axis limits were set as the 1st percentile and the 99th percentiles.*

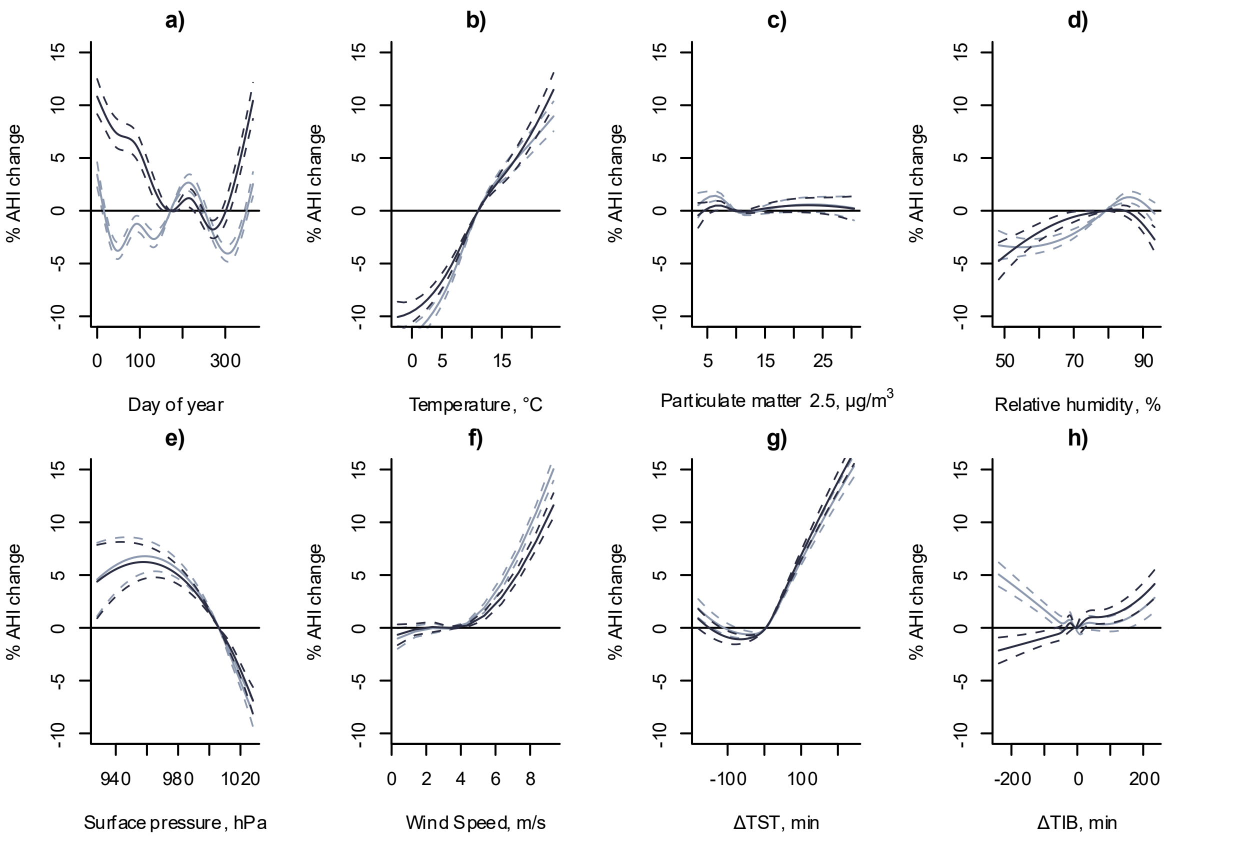

Figure SC11: Belgium.

Model description: *Associations of different environmental and sleep-specific factors with seasonal variation in the apnoea-hypopnoea-index (AHI) for unadjusted (blue) and fully adjusted models (black). a) Day of the year (21st of June as reference), b) 24h average temperature, c) density of particulate matter with diameter of less than 2.5um d) relative humidity e) surface pressure, f) wind speed g) difference between a given night total sleep time (TST) with the yearly TST average (ΔTST) in minutes and h) similar to g) but for time in bed (ΔTIB). All graphs represent estimated marginal means using the 50th percentiles as the reference value (except for a)), and the x-axis limits were set as the 1st percentile and the 99th percentiles.*

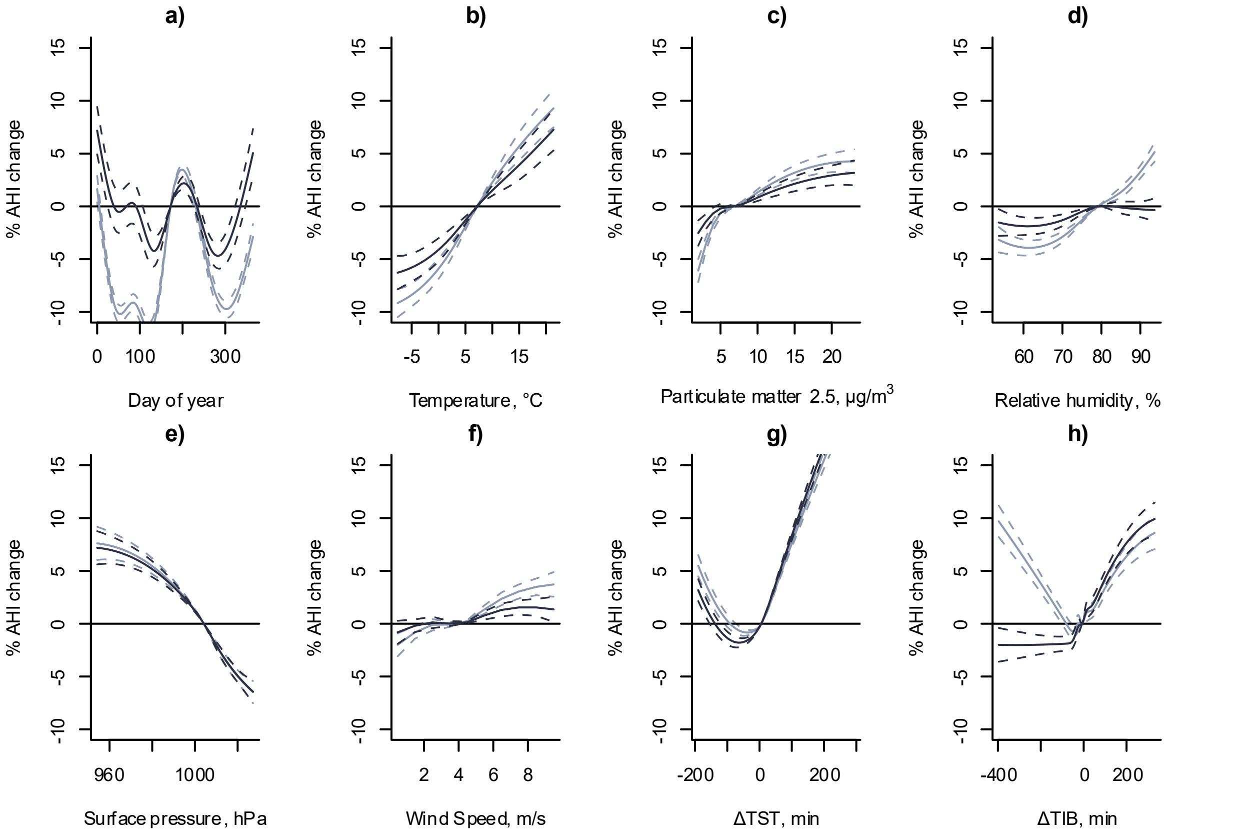

Figure SC12: Sweden.

Model description: *Associations of different environmental and sleep-specific factors with seasonal variation in the apnoea-hypopnoea-index (AHI) for unadjusted (blue) and fully adjusted models (black). a) Day of the year (21st of June as reference), b) 24h average temperature, c) density of particulate matter with diameter of less than 2.5um d) relative humidity e) surface pressure, f) wind speed g) difference between a given night total sleep time (TST) with the yearly TST average (ΔTST) in minutes and h) similar to g) but for time in bed (ΔTIB). All graphs represent estimated marginal means using the 50th percentiles as the reference value (except for a)), and the x-axis limits were set as the 1st percentile and the 99th percentiles.*

Supplementary code

Main model specification can be seen below. The specification for the unadjusted model is the basis for Figure 2 and 3. Specific models were constructed following similar specification to investigate the association between environmental factors and AHI severity (Figure 4), which can be found in Table S1.

```r
dp$outcome <- 100*(dp$apnea_hypopnea_index-dp$yearly_AHI)/dp$yearly_AHI
dp$userid <- as.factor(dp$userid)
dp$year <- as.factor(dp$year)

dp$idfactor <- do.call(paste, c(dp[,c('userid','year')], sep="-"))
dp$idfactor <- as.factor(dp$idfactor)

#########################################################
# define spline variables
t2m <- onebasis(dp$t2m_mean, "ns", df=4)
tstm <- onebasis(dp$tst_diff, "ns", df=4)
rh <- onebasis(dp$relative_humidity, "ns", df=4)
tcc <- onebasis(dp$tcc_mean, "ns", df=4)
sp <- onebasis(dp$sp_mean, "ns", df=4)
pm <- onebasis(dp$pm2p5_mean, "ns", df=4)
doy <- onebasis(dp$day_of_year, "ns", df=8)
wss <- onebasis(dp$wind_speed, "ns", df=4)
tsb <- onebasis(dp$time_start_diff, "ns", df=8)

#####################################
## DOY
mdoy <- gnm(outcome ~ weekday + doy, data=dp, eliminate=factor(dp$idfactor))
doypredunadjusted <- crosspred(doy, mdoy, cen=172, at=0:365)

mdoy_fa <- gnm(outcome ~ t2m + weekday + rh + tcc + sp + pm+doy+tsb+tstm+wss, data=dp,
               eliminate=factor(dp$idfactor), trace=TRUE, verbose=TRUE)
doypred_fa <- crosspred(doy, mdoy_fa, cen=172, at=0:365)
```
