# Supplementary material for: Obstructive sleep apnea severity varies by season and environmental influences such as ambient temperature
Source: Commun Med (Lond). 2025 Jul 29;5:314. doi: 10.1038/s43856-025-01016-0 (PMC12307740; doi:10.1038/s43856-025-01016-0)
Supplement: Supplementary file 3 — Supplemental Data legends [file 43856_2025_1016_MOESM3_ESM.pdf]

Source data to reproduce Figures 2 and 3 are available in the “Supplemental data 1.xlsx”.

Exposure-response curve and Source data to reproduce Figure 4 is provided in the Supplemental data 2.rds file.
